# Supplementary material for: Mechanistic investigation of human maturation of Okazaki fragments reveals slow kinetics
Source: Nat Commun. 2022 Nov 15;13:6973. doi: 10.1038/s41467-022-34751-2 (PMC9666535; doi:10.1038/s41467-022-34751-2)
Supplement: Supplementary file 1 — Supplementary Information [file 41467_2022_34751_MOESM1_ESM.pdf]

Supplementary Information

Mechanistic investigation of human maturation of Okazaki fragments reveals slow kinetics

Vlad-Stefan Raducanu[1, #], Muhammad Tehseen[1, #], Amani Al-Amodi[1], Luay I. Joudeh[1], Alfredo De Biasio[1, *] and Samir M. Hamdan[1, *]

[1]Bioscience Program, Division of Biological and Environmental Sciences and Engineering, King Abdullah University of Science and Technology, Thuwal 23955, Saudi Arabia.

[#]These authors contributed equally.

*Correspondence: alfredo.debiasio@kaust.edu.sa, samir.hamdan@kaust.edu.sa

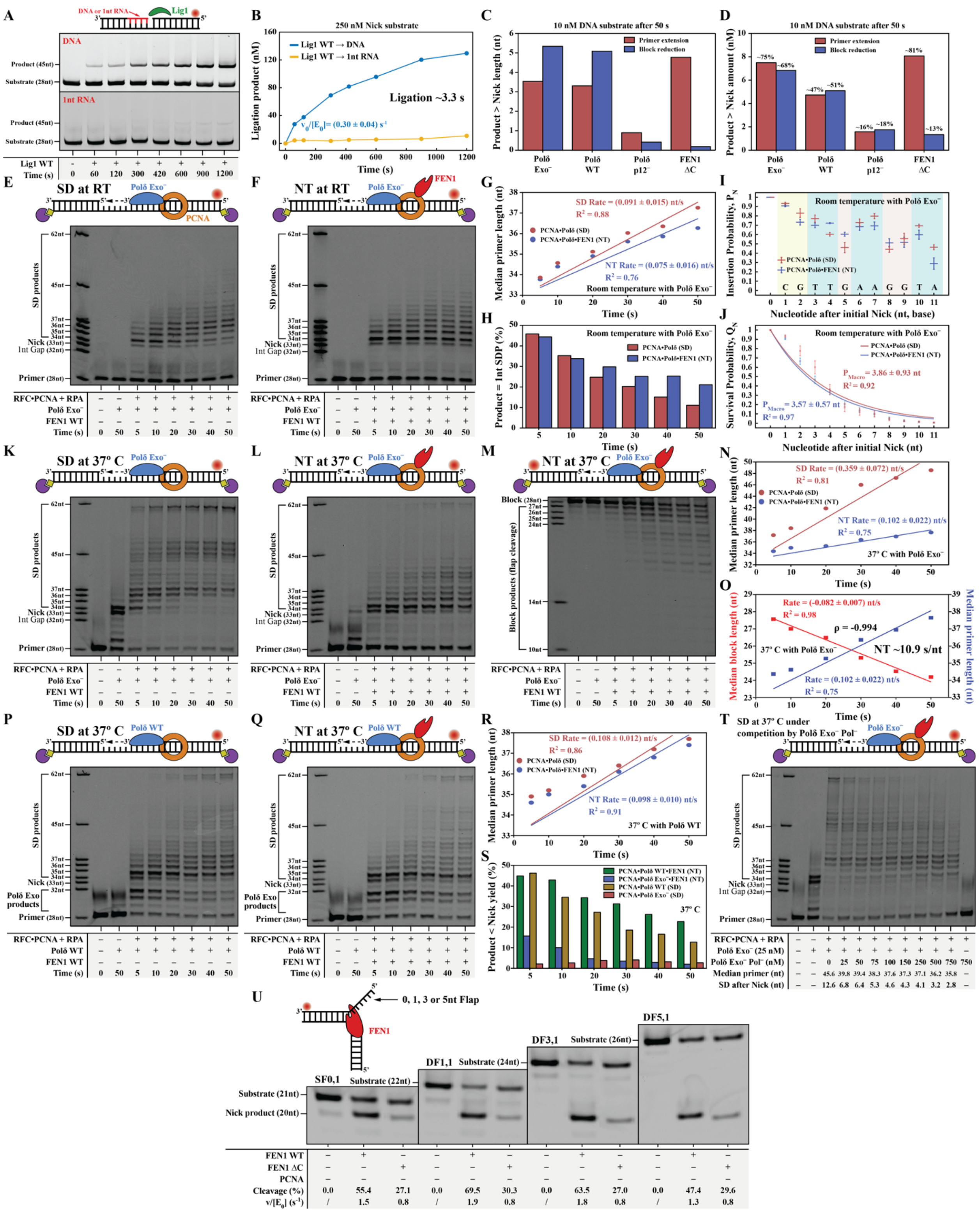

Supplementary Fig. 1. Reconstitution of the human NT and SD reactions. (A) Multiple turnover ligation assays of nick substrates containing a complete DNA block (Sub#5; Supplementary Fig. 7) or 1-nt RNA to the 5' end of the nick junction (Sub#6; Supplementary Fig. 7) under 1 nM Lig1 conditions. **(B)** Quantification of the ligation yields

of the reactions presented in panel A. For Lig1 WT, the initial reaction rate was estimated as the slope of a linear fit of the first three experimental datapoints. **(C)** and **(D)** Quantification of the NT **(C)** reaction median product lengths and **(D)** yields from Figures 1D, E. The quantification was performed for the reaction products formed in 50 s and the median length synthesis was determined using median analysis as described in Methods. NT products were only quantified for those above the length of the first NP. **(E)** SD and **(F)** NT products of a primer-labeled 5-nt gap (Sub#2; Supplementary Fig. 7) at room temperature (RT) under 25 nM Polδ Exo^{-} and 25 nM FEN1 conditions. **(G)** Quantification of SD (panel E) and NT (panel F) median product lengths as a function of time. Experimental datapoints were fitted to linear dependencies with a fixed intercept of 33 nt. **(H)** Quantification of the relative abundance of the 1-nt NT products at various intervals determined from panel F. **(I)** Quantification of the insertion probabilities of the first 11 nt following gap closure from the data presented in panels E and F. Nucleotide insertion probabilities were determined using Eq. S21 (Supplementary Methods). **(J)** Product survival probabilities estimated from the data presented in panel I using Eq. S22 (Supplementary Methods). The experimental datapoints were fitted to single-exponential survival functions as detailed in Eq. S26 (Supplementary Methods). For panels I and J the datapoints and the error bars represent the average and the standard deviation between the probabilities estimated at 30, 40 and 50 s reaction times. **(K)** SD products of a primer-labeled 5-nt gap (Sub#2), **(L)** NT products of a primer-labeled 5-nt gap (Sub#2), and **(M)** NT products of a block-labeled 5-nt gap (Sub#3; Supplementary Fig. 7) at 37 °C under 25 nM Polδ Exo^{-} and 25 nM FEN1 conditions. **(N)** Quantification of SD (panel K) and NT (panel L) median product lengths as a function of time. Experimental datapoints were fitted to linear dependencies with a fixed intercept of 33 nt. **(O)** Quantification of the reaction rates presented in panel L and M. Median product lengths beyond the first NP were determined for each reaction time as described in Methods. The experimental datapoints were fitted to linear dependencies with fixed intercepts (28 nt for block length reduction and 33 nt for primer length increase). **(P)** SD and **(Q)** NT products of a primer-labeled 5-nt gap (Sub#2) at 37 °C under 25 nM Polδ WT and 25 nM FEN1 conditions. **(R)** Quantification of SD (panel P) and NT (panel Q) median product lengths as a function of time. Experimental datapoints were fitted to linear dependencies with a fixed intercept of 33 nt. **(S)** Quantification of the yield of products with lengths strictly higher than the Primer (28 nt) and strictly lower than the first NP (33 nt) as a percentage of total products not including the substrate, from panels K, L, P, and Q. **(T)** SD products of a primer-labeled 5-nt gap (Sub#2) at 37 °C for 30 s under 25 nM Polδ Exo^{-} conditions in the presence of increasing concentration of Polδ Exo^{-} Pol^{-} as competitor for dissociated Polδ Exo^{-}. The reactions were initiated by the addition of dNTPs and Polδ Exo^{-} Pol^{-}. Median SD product lengths beyond the first NP were determined at each Polδ Exo^{-} Pol^{-} concentration as described in Methods. **(U)** Multiple turnover cleavage of flap substrates with various 5' flap lengths (Subs#7–10; Supplementary Fig. 7) by FEN1 WT or FEN1 ΔC in the absence of PCNA under the following conditions: 1 nM FEN1, 37 °C, 3 min. Source data are provided as a Source Data file.

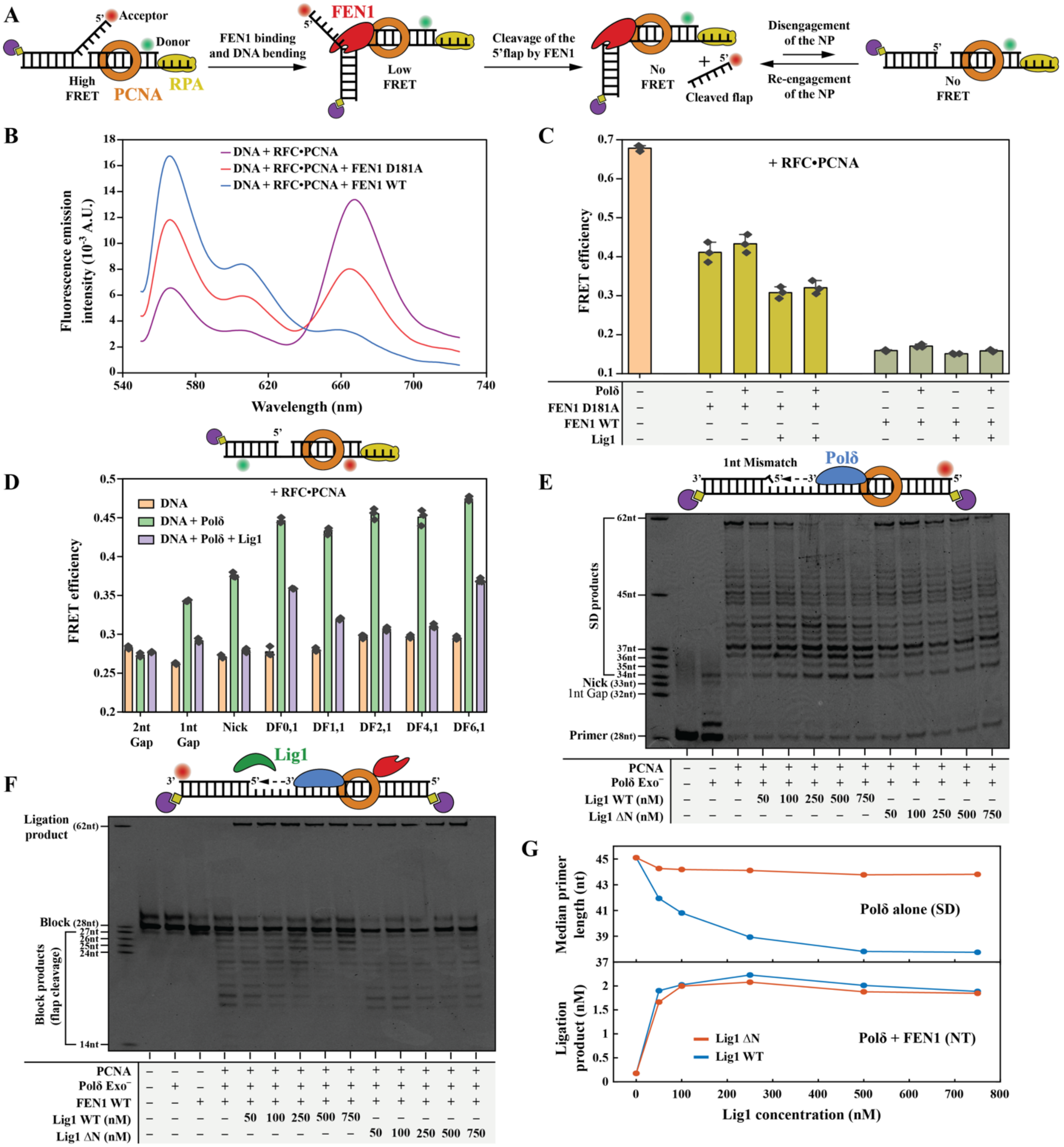

Supplementary Fig. 2. MOF protein interactions. (A) Schematic illustration of the flap labeling scheme used to monitor flap cleavage. **(B)** Examples of emission spectra of the flap-labeled double flap (Sub#22; Supplementary Fig. 7) after the addition of various MOF protein combinations. **(C)** Apparent FRET efficiencies of the flap-labeled double flap (Sub#22) after the addition of various MOF protein combinations in the presence of RFC•PCNA and under the following conditions: 15 nM FEN1, 250 nM Polδ, and 500 nM Lig1. The bar chart illustrates the mean (as bar height) and one standard deviation (as error bar) of three independent measurements. **(D)** Apparent FRET efficiencies of the internally labeled substrates (Subs#14–21; Supplementary Fig. 7) after the addition of

Polδ and Lig1. The bar chart illustrates the mean (as bar height) and one standard deviation (as error bar) of three independent measurements. **(E)** and **(F)** The effect of increasing the Lig1 WT or Lig1 ΔN concentrations on the **(E)** SD and **(F)** NT reactions. For panel E, the DNA substrate was a 5-nt gap containing an unphosphorylated and unpaired 5' nucleotide (Sub#4; Supplementary Fig. 7); while for panel F, an unmodified 5-nt gap was used (Sub#2; Supplementary Fig. 7) under the following conditions: 250 nM Polδ, 250 nM FEN1, RT, 30 seconds. **(G)** Quantification of the experimental data presented in panels E (top) and F (bottom). The top sub-panel was generated using the median analysis on each lane for products above and including the first NP. Source data are provided as a Source Data file.

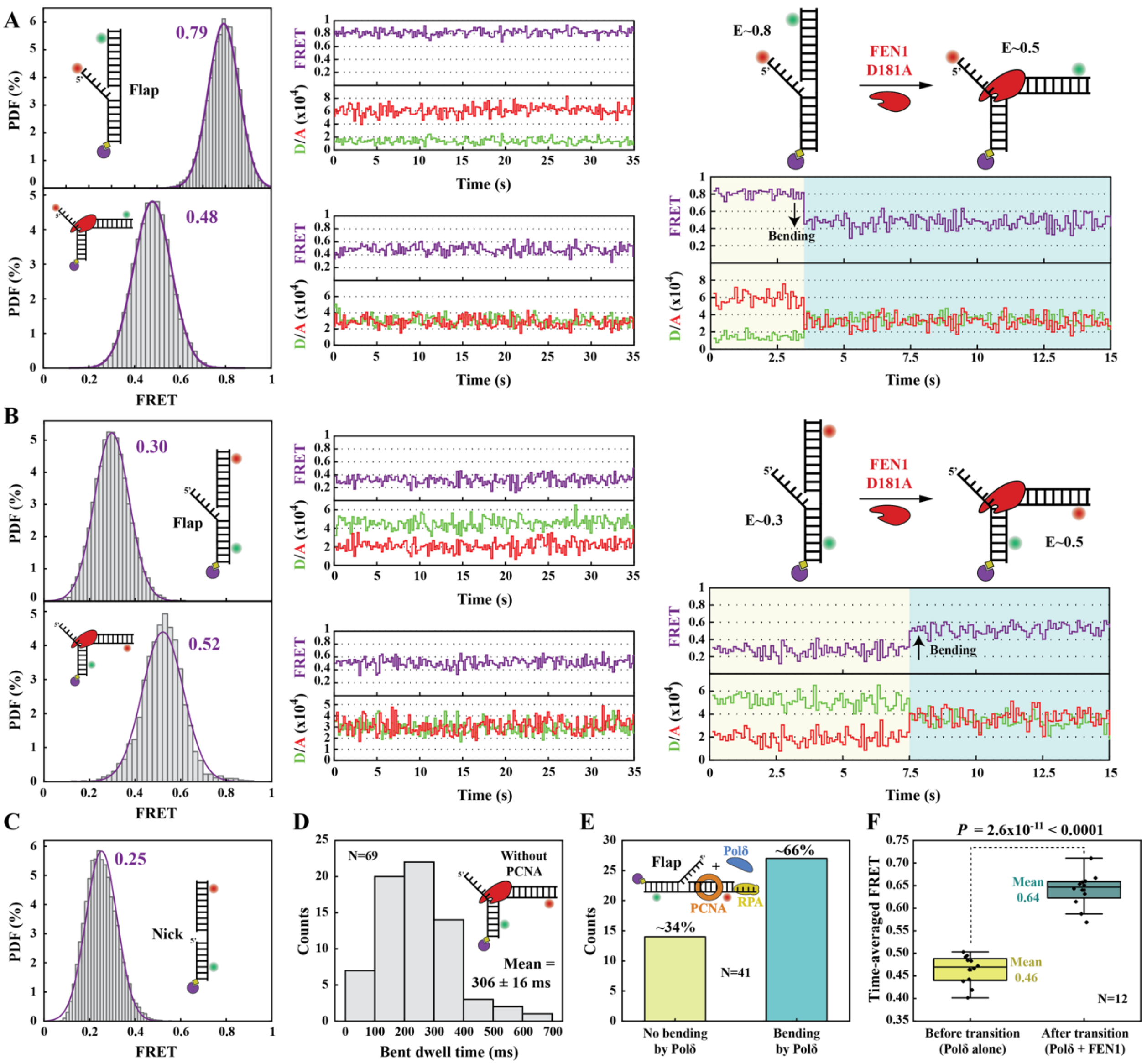

Supplementary Fig. 3. FEN1 and Polδ interactions monitored at the single-molecule level. (A) and **(B)** Monitoring FEN1 (250 nM FEN1 D181A)-induced bending at the single-molecule level through **(A)** the flap labeling scheme (Sub#22; Supplementary Fig. 7) and **(B)** the internal labeling scheme (Sub#12; Supplementary Fig. 7). In the absence of FEN1, the substrates were found in single stable FRET states. Upon FEN1 addition, a sharp FRET transition was observed. At saturating FEN1 concentration the substrates were found in different stable FRET states. All single-molecule time traces were acquired with a temporal resolution of 160 ms. **(C)** Histogram illustrating the distribution of FRET efficiencies of the NP (Sub#24; Supplementary Fig. 7) determined from single-molecule measurements. For all FRET states histograms, the average FRET efficiencies for the various substrates and conformations were obtained as the mean of histogram-fitted Gaussian distributions. **(D)** Histogram of the distributions of dwell times of the bent conformer from the internal labeling scheme in the absence (Sub#12) of pre-loaded PCNA from time-traces similar to the one illustrated in Figure 3D. The indicated mean

and error of the dwell time distribution represent the raw arithmetic mean and standard error of the mean of the raw datapoints that were binned into the histogram, without additional histogram fitting. **(E)** Bar chart illustrating the efficiency of Polδ holoenzyme assembly on the double flap (Sub#12) in the presence of pre-loaded PCNA as deduced from a single-molecule field of view (time-traces illustrated in Figures 3F, G). **(F)** Box plot illustrating the time-averaged FRET efficiencies before and after the transition of the double flap (Sub#12) from Polδ to FEN1 as deduced from time-traces similar to the one illustrated in Figure 3**I**. The box of the box plot is bounded by the 25th and 75th percentiles, and the centerline is placed at the median level (50th percentile). The whiskers of the box plot are placed at the 5th and 95th percentiles. The experimental datapoints, spanning from the minimum to the maximum values, are overlapped over the boxes. The *p*-value was determined using a two-sample two-sided *t*-test for equal means together with unknown and unequal standard deviations (a Behrens-Fisher problem). The MATLAB implementation of the test uses Satterthwaite's approximation for the effective degrees of freedom. No additional adjustments were applied to the data or to the test. Source data are provided as a Source Data file.

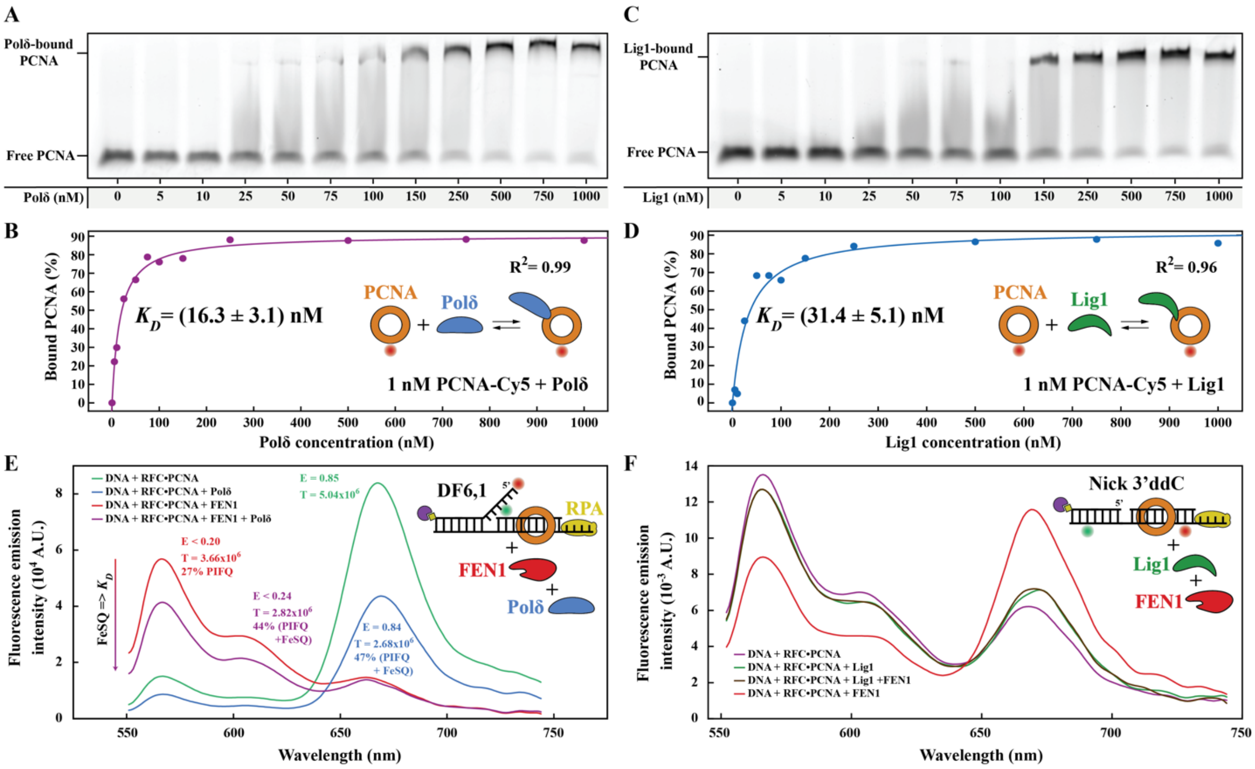

Supplementary Fig. 4. MOF protein interactions via PCNA. (A)–(D) Protein-protein EMSA to test the interaction between **(A)** Polδ and **(C)** Lig1 using PCNA in solution in the absence of DNA. Quantification of the experimental results illustrated in **(B)** panel A and **(D)** panel C. The experimental datapoints were fitted to a dependence proportional to that in Eq.1 (Methods). **(E)** Emission spectra of a double flap (Sub#25; Supplementary Fig. 7) labeled with Cy5 at the tip of the 5'flap and Cy3 at position four downstream of the 3' end of the double flap junction in the presence of NT proteins. This experiment, conducted under 250 nM Polδ and 15 nM FEN1 conditions, monitored both the flap cleavage induced by FEN1 (FRET and PIFQ) as well as the recruitment of Polδ (FeSQ) to form the FEN1•Polδ•PCNA toolbelt complex on the FEN1 cleavage-generated NP: 250 nM Polδ, 15 nM FEN1. **(F)** Emission spectra of an internally labeled NP (Sub#26; Supplementary Fig. 7) in the presence of FEN1 (2000 nM), pre-loaded PCNA, and/or Lig1 (500 nM). Source data are provided as a Source Data file.

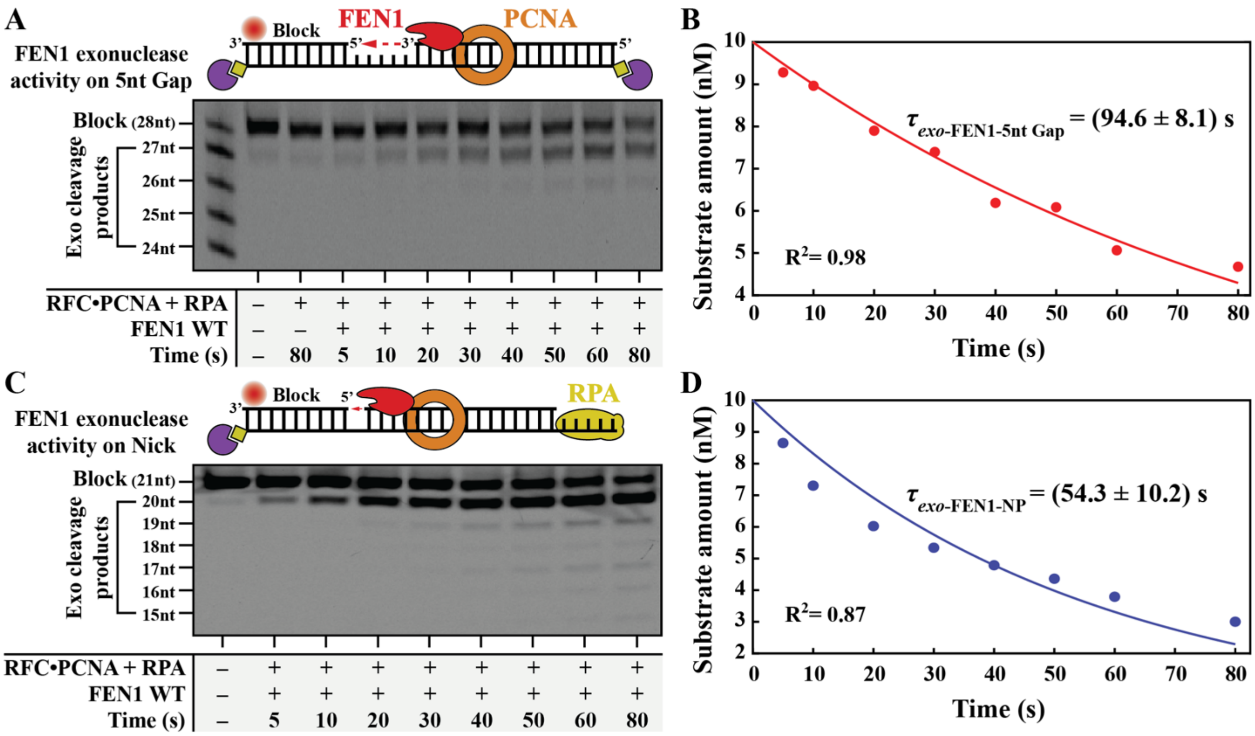

Supplementary Fig. 5. FEN1 exonuclease activities. (A) and **(C)** FEN1 (250 nM) exonuclease activity on **(A)** a 5-nt gap (Sub#3; Supplementary Fig. 7) and **(C)** NP (Sub#11; Supplementary Fig. 7), in the presence of pre-loaded PCNA. **(B)** and **(D)** Quantification of the time-dependence of FEN1 exonuclease activity illustrated in **(B)** panel A and **(D)** panel C. The experimental datapoints were fitted to single-exponential substrate-depletion burst equations as $\left[Substrate\ amount = 10\ \mathrm{nM} * e^{-t/\tau_{exo-\mathrm{FEN1-5nt\ Gap}}}\right]$ and $\left[\mathrm{Substrate\ amount} = 10\ \mathrm{nM} * e^{-t/\tau_{exo-\mathrm{FEN1-NP}}}\right]$. Source data are provided as a Source Data file.

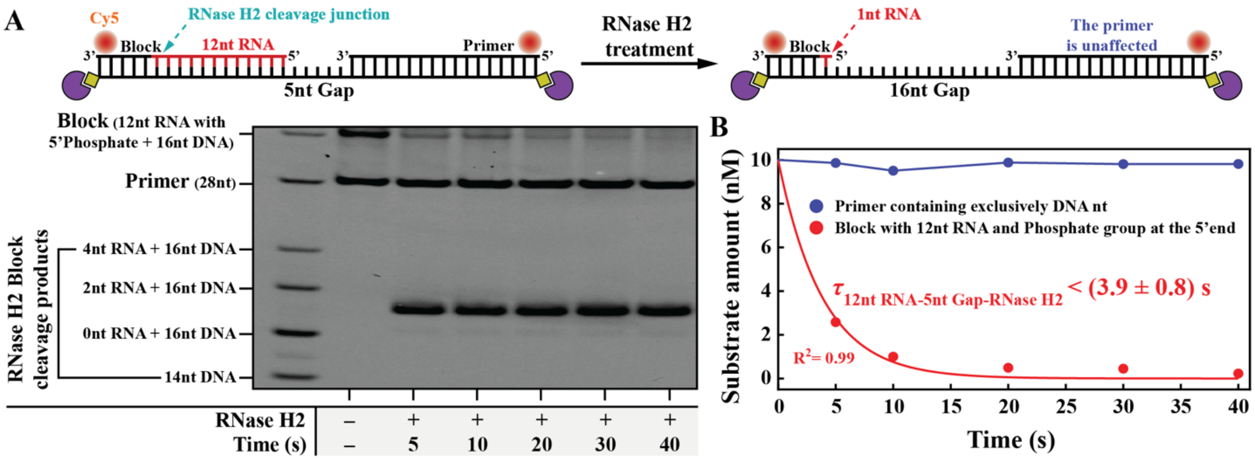

Supplementary Fig. 6. RNase H2 ribonuclease activity. (A) RNase H2 (250 nM) cleavage activity on a 5-nt gap (Sub#33; Supplementary Fig. 7) containing 12-nt RNA at the 5' end of the block. **(B)** Quantification of the time-dependence of RNase H2 exonuclease activity is presented in panel A. The experimental block reduction datapoints were fitted to a single-exponential substrate-depletion burst equation as $\left[\text{Substrate amount} = 10\ \text{nM} * e^{-t/\tau_{\text{12nt RNA}-\text{5nt Gap}-\text{RNase H2}}}\right]$. Source data are provided as a Source Data file.

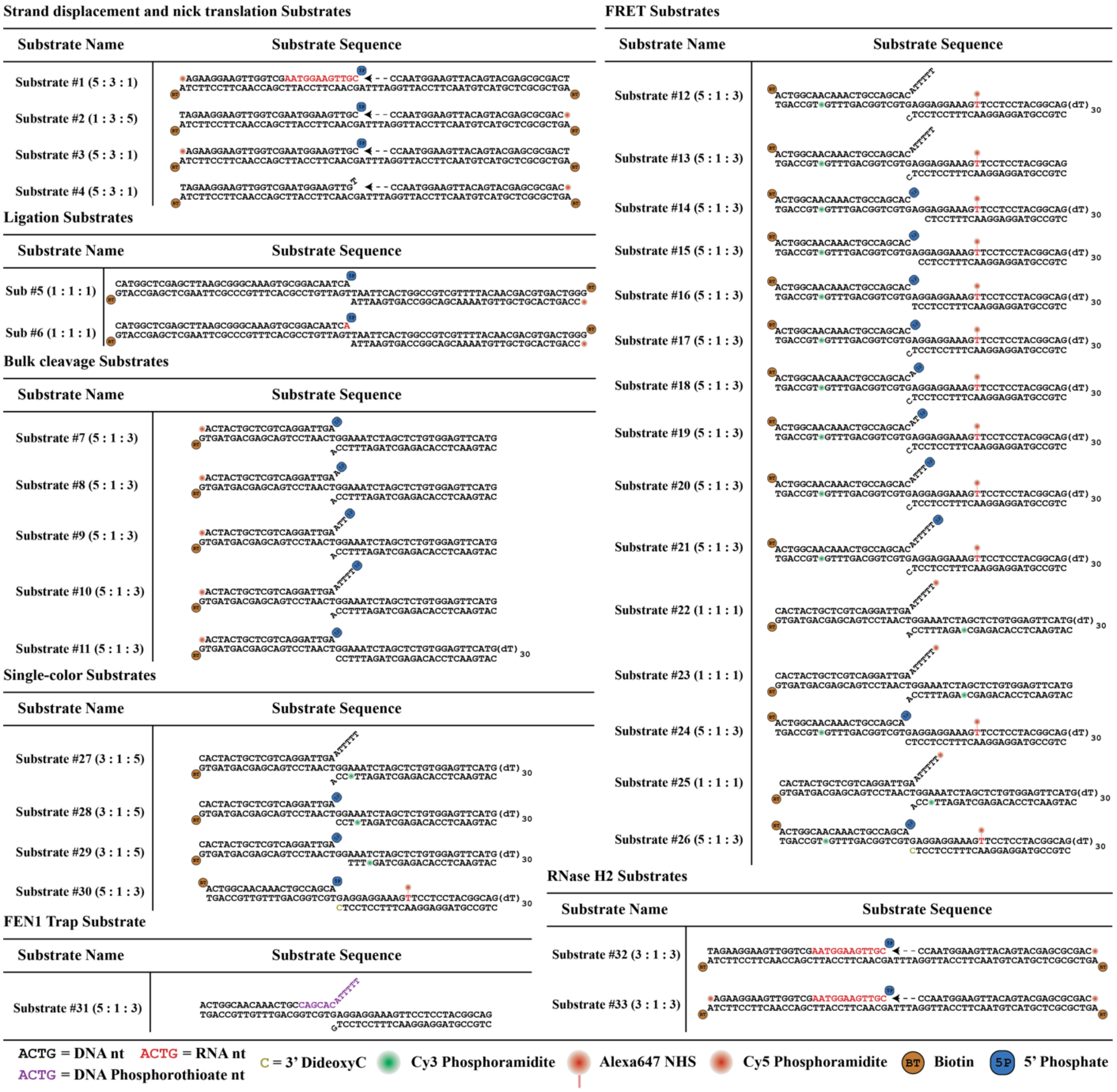

Supplementary Fig. 7. Structure and sequences of the DNA substrates used. The mixing ratios of the oligonucleotides used for substrate annealing before gel purification are indicated as 3'Primer:Template:5'Block/Flap ratios, with 3' and 5' relative to the junction.

Supplementary Methods

Derivation of a fitting model for polymerization kinetics data

The purpose of the current derivation is to obtain and prove analytical expressions that relate well-established processivity parameters to the empirical cumulative occupancies of polymerization products (Eqs. 19, 21, 25, 26). These equations are employed for the empirical data analysis presented in Figures S1I, J. The kinetic scheme used for the derivation is based on the model presented in Ref.[1] for a DNA motor moving in uniform steps with finite processivity. The scheme is expanded to include the contribution of protein-unbound intermediate products to the empirical cumulative occupancies of the polymerization products, which are not taken into account in the original model presented in Ref.[1]. In general, throughout the existing literature, the expression illustrated by Eq. S21 is consider well-established and employed empirically without additional proof based on an underlying kinetic model.

Consider the following: a DNA polymerase enzyme (*E*) associates with a primer-template DNA substrate (S_0) prior to the initiation of the polymerization reaction to give an enzyme-substrate complex (ES_0). The efficiency of the assembly of this complex is dictated by the enzyme and substrate concentrations as well as their affinity. Nevertheless, for all intents and purposes, a case is considered here in which this initial complex formation is saturated. Upon the initiation of the polymerization reaction, for example, by the addition of dNTPs, the chain reaction can be modeled via the following kinetic scheme:

$$\begin{array}{ccccccccccccccc} ES_0 & \overset{k_f}{\rightarrow} & ES_1 & \overset{k_f}{\rightarrow} & ES_2 & \overset{k_f}{\rightarrow} & \dots & \overset{k_f}{\rightarrow} & ES_N & \overset{k_f}{\rightarrow} & \dots & \overset{k_f}{\rightarrow} & ES_{N_{max}-1} & \overset{k_f}{\rightarrow} & ES_{N_{max}} \\ \downarrow^{k_d} & & \downarrow^{k_d} & & \downarrow^{k_d} & & & & \downarrow^{k_d} & & & & \downarrow^{k_d} & & \\ S_0 & & S_1 & & S_2 & & & & S_N & & & & S_{N_{max}-1} & & \end{array} \quad (S1)$$

where *N* represents each substrate extension length from 0 to N_{max}, k_f is the rate of incorporation of the following nucleotide, k_d is the dissociation rate of the polymerase from the current substrate, ES_N is the polymerase-bound substrate extended by *N* nucleotides,

S_N is the substrate extended by N nucleotides after polymerase dissociation, and N_{max} is the maximum number of nucleotides that can be added by polymerase because of the finite length of the DNA template. This scheme is precise in instances where a trap is added to prevent the re-binding of free polymerases to the S_N intermediates. Should a trap not be present, then k_d would be the net rate of dissociation, defined as the difference between the true rate of dissociation and the instantaneous rate of enzyme re-association. The fractional occupancy of each of the ES_N intermediates for such an N-steps kinetic scheme was derived previously[1] as:

$$\begin{cases} f_{ES_N}(t) = \frac{\left(k_f t\right)^N}{\Gamma(N+1)} e^{-(k_f+k_d)t}, \ for\ 0 \leq N < N_{max} \\ f_{ES_{N_{max}}}(t) = \left(\frac{k_f}{k_f + k_d}\right)^{N_{max}} \frac{\gamma\left(N_{max}, \left(k_f + k_d\right)t\right)}{\Gamma(N_{max})} \end{cases} \quad (S2)$$

where $\Gamma(N)$ is the Euler Gamma function defined as $\Gamma(N) = \int_0^\infty x^{N-1}e^{-x}dx$ and $\gamma(N,z)$ is the lower incomplete gamma function defined as $\gamma(N,z) = \int_0^z x^{N-1}e^{-x}dx$ with a dummy integration variable x. Upon reaction quenching, proteinase treatment, and substrate denaturation, both ES_N and S_N intermediates contribute to the intensity of the same N^{th} product band; therefore, the expression of the fractional occupancy of the S_N intermediates needs to be derived. This procedure starts by noting the kinetic equation for S_N formation:

$$\frac{d[S_N](t)}{dt} = k_d\ [ES_N](t) \quad (S3)$$

where square brackets denote the concentrations of the corresponding species. Dividing both sides by the total substrate concentration allows for Eq. S3 to be formed with fractional occupancies:

$$\frac{df_{S_N}(t)}{dt} = k_d\ f_{ES_N}(t) \quad (S4)$$

This final equation can be integrated directly, giving the fractional occupancy of the substrate extended by *N* nucleotides following polymerase dissociation:

$$f_{S_N}(t) = k_d \int_0^t f_{ES_N}(x)dx = \frac{k_d\, {k_f}^N}{\left(k_f + k_d\right)^{N+1}} \frac{\gamma\left(N+1, \left(k_f + k_d\right)t\right)}{\Gamma(N+1)}, \quad for\ 0 \le N < N_{max} \quad (S5)$$

As mentioned previously, both ES_N and S_N intermediates contribute to the intensity of the same N^{th} product band; therefore, the fractional intensity of the Nth product band is obtained as the sum of $f_{ES_N}(t)$ and $f_{S_N}(t)$ which is denoted as $f_N(t)$ with the general expression:

$$\begin{cases} f_N(t) = \dfrac{\left(k_f t\right)^N}{\Gamma(N+1)} e^{-(k_f + k_d)t} + \dfrac{k_d\, {k_f}^N}{\left(k_f + k_d\right)^{N+1}} \dfrac{\gamma\left(N+1, \left(k_f + k_d\right)t\right)}{\Gamma(N+1)}, \quad for\ 0 \le N < N_{max} \\ f_{N_{max}}(t) = \left(\dfrac{k_f}{k_f + k_d}\right)^{N_{max}} \dfrac{\gamma\left(N_{max}, \left(k_f + k_d\right)t\right)}{\Gamma(N_{max})} \end{cases} \quad (S6)$$

To simplify these expressions, the following variable changes can be made, as was previously described[1]:

$$\begin{cases} k_{obs} = k_f + k_d \\ P = \dfrac{k_f}{k_f + k_d} \end{cases} \Leftrightarrow \begin{cases} k_f = P\, k_{obs} \\ k_d = (1-P)\, k_{obs} \end{cases} \quad (S7)$$

Given these changes, the system described in Eq. S6 can be redefined as:

$$\begin{cases} f_N(t) = P^N \dfrac{(k_{obs}\, t)^N}{\Gamma(N+1)} e^{-k_{obs}\, t} + P^N (1-P) \dfrac{\gamma(N+1, k_{obs}\, t)}{\Gamma(N+1)}, \quad for\ 0 \le N < N_{max} \\ f_{N_{max}}(t) = P^{N_{max}} \dfrac{\gamma(N_{max}, k_{obs}\, t)}{\Gamma(N_{max})} \end{cases} \quad (S8)$$

where k_{obs} is the effective reaction rate, and *P* is a microscopic factor related to enzyme processivity[1,2]. At a fixed reaction time, $f_N(t)$ can be regarded as a discrete probability momentum function (PMF) of the distribution of various intermediate products (e.g., the distribution of normalized band intensities on a gel). This PMF system contains all the kinetic information about the system. Since the Gamma functions are well defined, even for real-valued *N*, this PMF can be naturally extended to a continuous probability momentum function (PDF). Nevertheless, for model fitting purposes, in general, the cumulative density function (CDF) functions better than the PDF, as integration may average random fluctuations. Therefore, we define the cumulative density of the products from *0* to *N* (e.g., for data obtained by the integration of intensity along a gel's lane, as was previously described for median rate determination[3]) as $F_N(t)$:

$$F_N(t) = \sum_{i=0}^{N} f_{ES_i}(t) + \sum_{i=0}^{N} f_{S_i}(t) = \sum_{i=0}^{N} f_i(t)\,,\ \ for\ 0 \le N < N_{max} \quad (S9)$$

Thereafter, the system can be redefined using Eq. S7 and the recurrence relations for the Gamma functions: $\Gamma(N+1) = N\,\Gamma(N)$ and $\gamma(N+1,z) = N\,\gamma(N,z) - z^N e^{-z}$. Following simple algebraic operations, the system can be redefined as:

$$\begin{cases} f_N(t) = P^N \dfrac{\gamma(N, k_{obs}\,t)}{\Gamma(N)} - P^{N+1} \dfrac{\gamma(N+1, k_{obs}\,t)}{\Gamma(N+1)},\ \ for\ 0 \le N < N_{max} \\ f_{N_{max}}(t) = P^{N_{max}} \dfrac{\gamma(N_{max}, k_{obs}\,t)}{\Gamma(N_{max})} \end{cases} \quad (S10)$$

The summation in Eq. S9 can then be carried out immediately as a telescoping series, yielding:

$$F_N(t) = P^0 \frac{\gamma(0, k_{obs}\,t)}{\Gamma(0)} - P^{N+1} \frac{\gamma(N+1, k_{obs}\,t)}{\Gamma(N+1)},\ \ for\ 0 \le N < N_{max} \quad (S11)$$

It is worth noting that the expression on the right-hand side is not directly well defined because the integrals that define the Gamma functions are not convergent for *N = 0*.

Nevertheless, this can be overcome by introducing a limit and by considering the following series expansion of the lower incomplete Gamma function: $\gamma(N,z) = z^N\, e^{-z}\, \Gamma(N)\, \sum_{k=0}^{\infty} \frac{z^k}{\Gamma(N+k+1)}$. Rearranging the terms and setting the limit to zero with respect to N yields results in the following for any finite non-zero z:

$$\lim_{N\to 0} \frac{\gamma(N,z)}{\Gamma(N)} = \lim_{N\to 0}\left(z^N\, e^{-z} \sum_{k=0}^{\infty} \frac{z^k}{\Gamma(N+k+1)}\right) = e^{-z} \sum_{k=0}^{\infty} \frac{z^k}{\Gamma(k+1)} = e^{-z} \sum_{k=0}^{\infty} \frac{z^k}{\mathrm{k!}} \quad (S12)$$

The last infinite sum is immediately recognized as the Maclaurin series expansion of e^z. With this observation, the above limit can be defined as:

$$\lim_{N\to 0} \frac{\gamma(N,z)}{\Gamma(N)} = e^{-z}\, e^{z} = e^0 = 1, \qquad \forall\, z \in \mathbb{R}^+ \quad (S13)$$

This observation defines the final expression for cumulative occupancy as:

$$F_N(t) = 1 - P^{N+1} \frac{\gamma(N+1, k_{obs}\, t)}{\Gamma(N+1)}, \;\; for\; 0 \le N < N_{max} \quad (S14)$$

Given that the fractional occupancies must add up to unity, as expected, it can easily be verified that $F_{N_{max}}(t) = F_{N_{max}-1}(t) + f_{N_{max}}(t) \equiv 1$. As such, the final kinetic model in cumulative form can be defined as:

$$\begin{cases} F_N(t) = 1 - P^{N+1} \dfrac{\gamma(N+1, k_{obs}\, t)}{\Gamma(N+1)}, \;\; for\; 0 \le N < N_{max} \\ F_{N_{max}}(t) = 1 \end{cases} \quad (S15)$$

Observations:

1. **Asymptotic behavior of the cumulative form in the long timescale, as $t \gg 1/k_{obs}$.** To determine this, the following property of the incomplete gamma function was used:

$\lim_{z\to\infty} \frac{\gamma(N,z)}{\Gamma(N)} = 1$, for any real finite *N*. If k_{obs} is finite and strictly positive, the desired limit can be defined as:

$$\lim_{t\to\infty} F_N(t) = 1 - P^{N+1} \lim_{t\to\infty} \frac{\gamma(N+1, k_{obs}\, t)}{\Gamma(N+1)} = 1 - P^{N+1},\ \ for\ 0 \le N < N_{max} \quad (S16)$$

This equation demonstrates that over a long timescale, the cumulative distribution of the products depends exclusively on the processivity.

2. **Concentration dependence of processivity in cases where enzyme re-association occurs.** For this purpose, the general step of the scheme presented in Eq. S1 can be reconsidered as:

$$\begin{matrix} \ldots & \overset{k_f}{\rightarrow} & ES_N & \overset{k_f}{\rightarrow} & \ldots \\ & & \uparrow^{c_E * k_{on}} \downarrow^{k_{off}} & & \\ & & S_N & & \end{matrix} \quad \sim \quad \begin{matrix} \ldots & \overset{k_f}{\rightarrow} & ES_N & \overset{k_f}{\rightarrow} & \ldots \\ & & \downarrow^{k_d} & & \\ & & S_N & & \end{matrix} \quad (S17)$$

where k_{off} is the microscopic dissociation rate, k_{on} is the microscopic association rate, and c_E is the free enzyme concentration. Given that free enzyme is present in the system, it can be assumed that the instantaneous dissociation rate, k_d, depends on a combination of k_{off} and $c_E * k_{on}$. Given that the processivity factor (i.e., Eq. S7) depends on k_d, it follows that it will also depend on $c_E * k_{on}$ and therefore also on c_E. In combination with the asymptotic behavior described above, the enzyme concentration dependence of the processivity can be used to assess whether complete dissociation and re-association of the enzyme takes place during the entirety of the intermediary steps. If processivity-like behavior is present on a long timescale, but it is concentration independent, then other explanations must be sought; such models may include bifurcations into inactive conformers, incomplete dissociation, and a non-uniform rate throughout the reaction. These intermediates may contribute to protein removal and substrate denaturation during the distribution of S_N.

3. **An alternative empirical derivation of processivity and an extension to pointwise processivity factors.** Inspired by methodology applied previously[4], the following limit can be considered:

$$\lim_{t\to\infty}\frac{\sum_{i=N+1}^{N_{max}} f_i(t)}{\sum_{i=N}^{N_{max}} f_i(t)} = \lim_{t\to\infty}\frac{\sum_{i=0}^{N_{max}} f_i(t) - \sum_{i=0}^{N} f_i(t)}{\sum_{i=0}^{N_{max}} f_i(t) - \sum_{i=0}^{N-1} f_i(t)} = \lim_{t\to\infty}\frac{1-\sum_{i=0}^{N} f_i(t)}{1-\sum_{i=0}^{N-1} f_i(t)} = \lim_{t\to\infty}\frac{1-F_N(t)}{1-F_{N-1}(t)} \quad (S18)$$

Considering the asymptotic behavior presented in Eq. S16, the desired limit can be redefined as:

$$\lim_{t\to\infty}\frac{\sum_{i=N+1}^{N_{max}} f_i(t)}{\sum_{i=N}^{N_{max}} f_i(t)} = \frac{1-1+P^{N+1}}{1-1+P^{N}} = P \quad (S19)$$

This expression offers an empirical method for determining the processivity factor from the relative distribution of the reaction products. In cases where the processivity is non-uniform throughout the reaction, as previously described[4], this expression is generalized as:

$$P_{N+1} = \lim_{t\to\infty}\frac{\sum_{i=N+1}^{N_{max}} f_i(t)}{\sum_{i=N}^{N_{max}} f_i(t)},\ \ for\ 0 \le N < N_{max} \quad (S20)$$

Moving the *N+1* index back by one unit on both sides of the equation above is useful, resulting in the following:

$$P_{N} = \lim_{t\to\infty}\frac{\sum_{i=N}^{N_{max}} f_i(t)}{\sum_{i=N-1}^{N_{max}} f_i(t)},\ \ for\ 1 \le N \le N_{max} \quad (S21)$$

Here, P_N describes an apparent pointwise processivity factor associated with the probability of the Nth product being formed over a long timescale. In other words, it describes the probability of moving forward from the $(N-1)^{th}$ step to the Nth step of the reaction. This value is considered apparent because the condition $t \to \infty$ is impossible to

achieve experimentally; moreover, some residual contribution of the rate may still be present, despite experimental efforts. By default, P_0 = 1 because the existence of ES_0 and S_0 does not involve any catalytic step. It is worth noting that mathematically, P_N describes a series of conditional probabilities because the Nth step of the reaction can only occur if all the previous *N-1* steps have already occurred. Based on the definition of conditional probability, the total probability of the formation of the Nth product can now be defined as:

$$Q_N = \prod_{i=0}^{N} P_i\,, \;\; for\; 0 \leq N \leq N_{max} \quad (S22)$$

Given the above definition of P_0, it can immediately be deduced that Q_0 = 1. For simple cases where the processivity factor is uniform throughout the reaction (i.e., P_i = P), then the last equation can be reduced to:

$$Q_N = P^N, \;\; for\; 0 \leq N \leq N_{max} \quad (S23)$$

Given Eq. S16, it is clear that $P^N = 1 - \lim_{t\to\infty} F_{N-1}(t)$. Inserting this expression on the right-hand side of Eq. S23 results in $Q_N = 1 - \lim_{t\to\infty} F_{N-1}(t)$. This expression alludes to the link between a survival function and its corresponding CDF in general. In fact, by following a few clear algebraic operations and considering that $0 < P \leq 1 < e$, it can be shown that:

$$Q_N = P^N = \left(e^{\ln P}\right)^N = \; e^{\mathrm{N}\ln P} = e^{-\mathrm{N}\ln\frac{1}{P}} \quad (S24)$$

The expression $e^{-\lambda N}$ can immediately be recognized as the survival function of an exponential distribution with a mean $1/\lambda$. Setting $\lambda = \ln\frac{1}{P}$ results in a mean macroscopic processivity defined as:

$$P_{Macro} = \frac{1}{\ln \frac{1}{P}} = -\frac{1}{\ln P} \quad (S25)$$

As such, the expression in Eq. S24 can finally be redefined as:

$$Q_N = e^{-N/P_{Macro}} \quad (S26)$$

An exponential decay fit of Q_N vs. N can immediately recover P_{Macro} in the same units as N. Further, as mentioned above, the intercept of the fit was $Q_0 = 1$. Ultimately, this mean macroscopic processivity (Eq. S25) contains less information than the set of pointwise processivity factors (Eq. S21), as it averages along the reaction steps. Nevertheless, the mean processivity can be useful for performing simple semi-quantitative comparisons under different reaction conditions.

Supplementary References

1 Lucius, A. L., Maluf, N. K., Fischer, C. J. & Lohman, T. M. General methods for analysis of sequential "n-step" kinetic mechanisms: application to single turnover kinetics of helicase-catalyzed DNA unwinding. *Biophys J* **85**, 2224-2239, doi:10.1016/S0006-3495(03)74648-7 (2003).

2 McClure, W. R. & Chow, Y. The kinetics and processivity of nucleic acid polymerases. *Methods Enzymol* **64**, 277-297, doi:10.1016/s0076-6879(80)64013-0 (1980).

3 Stodola, J. L. & Burgers, P. M. Resolving individual steps of Okazaki-fragment maturation at a millisecond timescale. *Nat Struct Mol Biol* **23**, 402-408, doi:10.1038/nsmb.3207 (2016).

4 Hedglin, M., Pandey, B. & Benkovic, S. J. Stability of the human polymerase delta holoenzyme and its implications in lagging strand DNA synthesis. *Proc Natl Acad Sci U S A* **113**, E1777-1786, doi:10.1073/pnas.1523653113 (2016).
