# Supplementary material for: Mechanistic investigation of human maturation of Okazaki fragments reveals slow kinetics
Source: Nat Commun. 2022 Nov 15;13:6973. doi: 10.1038/s41467-022-34751-2 (PMC9666535; doi:10.1038/s41467-022-34751-2)
Supplement: Supplementary file 3 — Reporting Summary [file 41467_2022_34751_MOESM3_ESM.pdf]

nature portfolio

Corresponding author(s): Alfredo De Biasio
Samir M. Hamdan

Last updated by author(s): Oct 28, 2022

Reporting Summary

Nature Portfolio wishes to improve the reproducibility of the work that we publish. This form provides structure for consistency and transparency in reporting. For further information on Nature Portfolio policies, see our Editorial Policies and the Editorial Policy Checklist.

Statistics

For all statistical analyses, confirm that the following items are present in the figure legend, table legend, main text, or Methods section.

n/a	Confirmed	
☐	☒	The exact sample size (*n*) for each experimental group/condition, given as a discrete number and unit of measurement
☐	☒	A statement on whether measurements were taken from distinct samples or whether the same sample was measured repeatedly
☐	☒	The statistical test(s) used AND whether they are one- or two-sided<br>*Only common tests should be described solely by name; describe more complex techniques in the Methods section.*
☒	☐	A description of all covariates tested
☒	☐	A description of any assumptions or corrections, such as tests of normality and adjustment for multiple comparisons
☐	☒	A full description of the statistical parameters including central tendency (e.g. means) or other basic estimates (e.g. regression coefficient) AND variation (e.g. standard deviation) or associated estimates of uncertainty (e.g. confidence intervals)
☐	☒	For null hypothesis testing, the test statistic (e.g. *F*, *t*, *r*) with confidence intervals, effect sizes, degrees of freedom and *P* value noted<br>*Give P values as exact values whenever suitable.*
☒	☐	For Bayesian analysis, information on the choice of priors and Markov chain Monte Carlo settings
☒	☐	For hierarchical and complex designs, identification of the appropriate level for tests and full reporting of outcomes
☒	☐	Estimates of effect sizes (e.g. Cohen's *d*, Pearson's *r*), indicating how they were calculated

Our web collection on statistics for biologists contains articles on many of the points above.

Software and code

Policy information about availability of computer code

Data collection	FluorEssence 3.5 (Horiba), xCellence rt 5.1 (Olympus)
Data analysis	OriginPro 2021b (OriginLab), MATLAB 2016b (MathWorks), MATLAB 2010a (MathWorks), TwoTone 3.1 (https://kapanidis.web.ox.ac.uk/software)

For manuscripts utilizing custom algorithms or software that are central to the research but not yet described in published literature, software must be made available to editors and reviewers. We strongly encourage code deposition in a community repository (e.g. GitHub). See the Nature Portfolio guidelines for submitting code & software for further information.

Data

Policy information about availability of data

All manuscripts must include a data availability statement. This statement should provide the following information, where applicable:

- Accession codes, unique identifiers, or web links for publicly available datasets
- A description of any restrictions on data availability
- For clinical datasets or third party data, please ensure that the statement adheres to our policy

All relevant data are included in the current manuscript and its supplementary information. Any additional information supporting the findings of this manuscript are available from the corresponding authors upon reasonable request. The source data underlying Figs. 1C, F; 2B, D; 3B, C, H; 4B, D; 5B, E and 6B, D and Supplementary Figs. 1B–D, G–J, N, O, R, S; 2C, D, G; 3D, F; 4B, D; 5B, D and 6B are provided as a Source Data file. The cellular expression levels of the Human MOF

proteins were estimated from the datasets presented in The Human Protein Atlas (proteinatlas.org) for Polδ p125 (ENSG00000062822), FEN1 (ENSG00000168496), and Lig1 (ENSG00000105486).

Human research participants

Policy information about studies involving human research participants and Sex and Gender in Research.

Reporting on sex and gender	N/A
Population characteristics	N/A
Recruitment	N/A
Ethics oversight	N/A

Note that full information on the approval of the study protocol must also be provided in the manuscript.

Field-specific reporting

Please select the one below that is the best fit for your research. If you are not sure, read the appropriate sections before making your selection.

☒ Life sciences ☐ Behavioural & social sciences ☐ Ecological, evolutionary & environmental sciences

For a reference copy of the document with all sections, see nature.com/documents/nr-reporting-summary-flat.pdf

Life sciences study design

All studies must disclose on these points even when the disclosure is negative.

Sample size	Sample size was not predetermined by any statistical method. For the fluorescence-based experiments, 3 individual measurements were performed, as, in general, very low variations (STD) were found due to the high stability of FRET and fluorescence modulation techniques.
Data exclusions	For the bulk experiments, no data were excluded. However, due to the nature of single-molecule experiments and photostability of fluorophores, a certain percentage of time traces showed premature photobleaching or excessive noise or aberrant FRET states, and therefore were excluded from the analysis of smFRET experiments (presented in Figures 3 and S3).
Replication	All measurements were repeated at least three times with reproducibility estimated at 95%. In general, the mean and standard deviation of the three replicates is provided. All bar graphs are overlaid with individual data points. For the single-molecule experiments, time traces were collected from three individual movies.
Randomization	The statistical randomization is not relevant to this study, as none of the data was assigned to particular sub-groups using statistical tools. All the assays performed in the current study require a rational approach for data categorization where applicable.
Blinding	Blinding does not apply to any of the biochemical or biophysical assays performed in the current study.

Reporting for specific materials, systems and methods

We require information from authors about some types of materials, experimental systems and methods used in many studies. Here, indicate whether each material, system or method listed is relevant to your study. If you are not sure if a list item applies to your research, read the appropriate section before selecting a response.

Materials & experimental systems

n/a	Involved in the study	
☒	☐	Antibodies
☒	☐	Eukaryotic cell lines
☒	☐	Palaeontology and archaeology
☒	☐	Animals and other organisms
☒	☐	Clinical data
☒	☐	Dual use research of concern

Methods

n/a	Involved in the study	
☒	☐	ChIP-seq
☒	☐	Flow cytometry
☒	☐	MRI-based neuroimaging
